# Short-term smoking increases the risk of insulin resistance

**DOI:** 10.1038/s41598-022-07626-1

**Published:** 2022-03-03

**Authors:** Soo Hyeon Cho, Sung Hoon Jeong, Jaeyong Shin, Sohee Park, Sung-In Jang

**Affiliations:** 1 Department of Public Health, Graduate School, Yonsei University, Seoul, Republic of Korea; 2 Institute of Health Services Research, Yonsei University, Seoul, Republic of Korea; 3 Department of Preventive Medicine and Institute of Health Services Research, Yonsei University College of Medicine, 50 Yonsei-ro, Seodaemun-gu, Seoul, 03722 Republic of Korea; 4 Department of Biostatistics, Yonsei University Graduate School of Public Health, Seoul, Republic of Korea

**Keywords:** Biomarkers, Diseases, Endocrinology, Health care, Health occupations

## Abstract

Insulin resistance can be affected directly or indirectly by smoking. This cross-sectional study aimed at examining the association between smoking patterns and insulin resistance using objective biomarkers. Data from 4043 participants sourced from the Korea National Health and Nutrition Examination Survey, conducted from 2016 to 2018, were examined. Short-term smoking patterns were used to classify participants according to urine levels of 4-(methylnitrosamino)-1-(3-pyridyl)-1-butanol and cotinine as continuous-smokers, past-smokers, current-smokers, and non-smokers. Insulin resistance was calculated using the triglyceride-glucose index from blood samples and was defined as either high or low. Multiple logistic regression analysis was performed to investigate the association between smoking behavior and insulin resistance. Men and women who were continuous-smokers (men: odds ratio [OR] = 1.74, *p* = 0.001; women: OR = 2.01, *p* = 0.001) and past-smokers (men: OR = 1.47, *p* = 0.033; women: OR = 1.37, *p* = 0.050) were more likely to have high insulin resistance than their non-smoking counterparts. Long-term smokers (≥ 40 days) are at an increased risk of insulin resistance in short-term smoking patterns. Smoking cessation may protect against insulin resistance. Therefore, first-time smokers should be educated about the health benefits of quitting smoking.

## Introduction

Insulin resistance is a growing metabolic disorder worldwide and is associated with some of the most common diseases affecting the modern society, including diabetes, high blood pressure, obesity, and coronary heart disease^1^. Direct methods of assessing insulin resistance include euglycemic-hyperinsulinemia clamp and insulin suppression tests and simple indirect indicators are estimated by the homeostasis model assessment of insulin resistance (HOMA-IR)^2–4^. However, these tests are invasive, complex, and expensive, making their application difficult in large-scale population studies and clinical practice^5^. Recently, the triglyceride and glucose (TyG) index, a simple and accurate marker of insulin resistance, has been proposed, which uses fasting triglyceride and blood glucose levels for calculation^6^. The TyG index can help screen people at high risk of diabetes mellitus with a simple blood test. Furthermore, studies using the TyG index in adults in the Republic of Korea showed that an increase in the TyG index was associated with an increase in the prevalence of coronary artery calcification or arterial stiffness^7,8^ and suggested that it is a useful tool for evaluating insulin resistance^6^.

Smoking is a lifestyle factor that may directly or indirectly affect insulin resistance^9^. Several prospective studies on the relationship between smoking and insulin resistance have shown that smoking is a risk factor for insulin resistance^10–13^. However, these studies have mostly used self-reporting as a method of measuring exposure to smoking, and this may have led to incorrect measurement, as self-reported and biomarker results show a consistency of only 46–53%; in addition, self-reports tend to be unreliable for quantitative assessments of smoking volume^14,15^. These findings suggest that an objective method of measuring smoking volume is required to account for the inherent bias in self-reported data.

Cotinine is the main metabolite of nicotine present in the blood, urine, hair, and saliva and is considered an indicator of exposure to nicotine smoke or current smoking^16^. While nicotine has a half-life of around 2 h in the blood, cotinine has a half-life of 18–24 h and reflects the accumulated exposure to environmental tobacco smoke^17^. In particular, urine cotinine levels may help determine the contribution of smoke in the air during the sampling process to the total smoking exposure^18^. 4-(methylnitrosamino)-1-(3-pyridyl)-1-butanone (NNAL) has been used extensively to assess the accuracy of self-reported smoking status^19,20^. NNAL is widely known as a biomarker of nicotine-derived nitrosamine ketone, a tobacco-specific lung carcinogen^21,22^. Furthermore, NNAL, due to its half-life of approximately 40 days, is useful for its tobacco specificity, association with carcinogen intake, facilitation of consistent detection of people exposed to tobacco, and evaluation of long-term exposure to harmful substances^23^.

Identifying an association between cotinine and NNAL, objective biomarkers of tobacco exposure, and insulin resistance may help assess the effect of smoking on the risk of insulin resistance. Although several studies based on self-reported data have been published regarding the influence of smoking on the risk of insulin resistance, to the best of our knowledge, no studies have examined this effect using objective smoking-related biomarkers. Therefore, this study investigated the relationship between smoking patterns and insulin resistance using cotinine and NNAL as biomarkers of tobacco exposure.

## Results

### Demographic characteristics

Of 4,043 participants, 2,067 (51.1%) were males (Table 1). Of the 2,067 male participants, 839 (40.6%), 454 (22.0%), 12 (0.6%), and 762 (36.9%) were continuous-smokers, past-smokers, current-smokers, and non-smokers, respectively. Of the 1976 (48.9%) female participants, 201 (10.2%), 452 (22.9%), 22 (1.1%), and 1301 (65.8%) were continuous-smokers, past-smokers, current-smokers, and non-smokers, respectively. The insulin resistance groups differed with respect to all factors except educational levels, household income, region, occupational categories, energy intake levels, secondhand smoking exposure, and the survey year.

**Table 1:** General characteristics of the study population.

Variables	Triglycerides and glucose index													
	Total		Men					Total		Women				
			Low IR Group (%)		High IR Group (%)		*P* value			Low IR Group (%)		High IR Group (%)		*P* value
	N	%	N	%	N	%		N	%	N	%	N	%	
**Total**	2067	100.0	610	29.5	1457	70.49		1976	100.0	1276	64.6	700	35.43	
**Short-term smoking pattern**							0.0003							0.0132
Continuous-smoker	839	40.6	206	33.8	633	43.4		201	10.2	112	8.8	89	12.7	
Past-smoker	454	22.0	140	23.0	314	21.6		452	22.9	285	22.3	167	23.9	
Current-smoker	12	0.6	3	0.5	9	0.6		22	1.1	12	0.9	10	1.4	
Non-smoker	762	36.9	261	42.8	501	34.4		1301	65.8	867	67.9	434	62.0	
**Age**							< 0.0001							< 0.0001
19–29	386	18.7	172	28.2	214	14.7		323	16.3	277	21.7	46	6.6	
30–39	410	19.8	123	20.2	287	19.7		347	17.6	258	20.2	89	12.7	
40–49	412	19.9	83	13.6	329	22.6		351	17.8	226	17.7	125	17.9	
50–59	352	17.0	78	12.8	274	18.8		399	20.2	232	18.2	167	23.9	
60–69	316	15.3	91	14.9	225	15.4		321	16.2	173	13.6	148	21.1	
≥ 70	191	9.2	63	10.3	128	8.8		235	11.9	110	8.6	125	17.9	
**Marital Status**							< 0.0001							0.0035
Married	1315	63.6	332	54.4	983	67.5		1245	63.0	774	60.7	471	67.3	
Single. widow, divorced, separated	752	36.4	278	45.6	474	32.5		731	37.0	502	39.3	229	32.7	
**Educational level**							0.1166							< 0.0001
Middle school or below	380	18.4	105	17.2	275	18.9		598	30.3	308	24.1	290	41.4	
High school	810	39.2	260	42.6	550	37.7		701	35.5	457	35.8	244	34.9	
College or over	877	42.4	245	40.2	632	43.4		677	34.3	511	40.0	166	23.7	
**Household income**							0.3308							< 0.0001
Low	290	14.0	97	15.9	193	13.2		356	18.0	187	14.7	169	24.1	
Mid-low	490	23.7	147	24.1	343	23.5		505	25.6	321	25.2	184	26.3	
Mid-high	618	29.9	170	27.9	448	30.7		563	28.5	375	29.4	188	26.9	
High	669	32.4	196	32.1	473	32.5		552	27.9	393	30.8	159	22.7	
**Region**							0.9638							0.5051
Urban area	1710	82.7	505	82.8	1205	82.7		1616	81.8	1049	82.2	567	81.0	
Rural area	357	17.3	105	17.2	252	17.3		360	18.2	227	17.8	133	19.0	
**Occupational categories**^**a**^							0.1170							< 0.0001
White	634	30.7	171	28.0	463	31.8		450	22.8	329	25.8	121	17.3	
Pink	224	10.8	58	9.5	166	11.4		338	17.1	230	18.0	108	15.4	
Blue	693	33.5	215	35.2	478	32.8		315	15.9	180	14.1	135	19.3	
Inoccupation	516	25.0	166	27.2	350	24.0		873	44.2	537	42.1	336	48.0	
**BMI**^**b**^							< 0.0001							< 0.0001
Underweight or Normal (< 25)	1253	60.6	460	75.4	793	54.4		1433	72.5	1038	81.3	395	56.4	
Overweight(≥ 25.0)	814	39.4	150	24.6	664	45.6		543	27.5	238	18.7	305	43.6	
**Drinking status**							0.0072							0.0040
No	284	13.7	103	16.9	181	12.4		609	30.8	365	28.6	244	34.9	
Yes	1783	86.3	507	83.1	1276	87.6		1367	69.2	911	71.4	456	65.1	
**Walking frequently**^**c**^							0.0071							0.0044
Inadequate	1023	49.5	274	44.9	749	51.4		1135	57.4	703	55.1	432	61.7	
Adequate	1044	50.5	336	55.1	708	48.6		841	42.6	573	44.9	268	38.3	
**Energy intake level**^**d**^							0.1847							0.8166
Inadequate	1242	60.1	380	62.3	862	59.2		1340	67.8	863	67.6	477	68.1	
Adequate	825	39.9	230	37.7	595	40.8		636	32.2	413	32.4	223	31.9	
**Chronic disease diagnosis**^**e**^							< 0.0001							< 0.0001
No	1570	76.0	499	81.8	1071	73.5		1457	73.7	1033	81.0	424	60.6	
Yes	497	24.0	111	18.2	386	26.5		519	26.3	243	19.0	276	39.4	
**Secondhand smoke exposure**							0.2078							0.1218
No	1354	65.5	412	67.5	942	64.7		1461	73.9	929	72.8	532	76.0	
Yes	713	34.5	198	32.5	515	35.3		515	26.1	347	27.2	168	24.0	
**Family history**^**f**^							0.0046							0.0510
No	1630	78.9	505	82.8	1125	77.2		1501	76.0	987	77.4	514	73.4	
Yes	437	21.1	105	17.2	332	22.8		475	24.0	289	22.6	186	26.6	
**Pack-Year of Smoking**							< 0.0001							0.0307
Pack-Years < 10	1150	55.6	402	65.9	748	51.3		1962	99.3	1248	97.8	670	95.7	
10 ≤ Pack-Years < 20	374	18.1	79	13.0	295	20.2		42	2.1	20	1.6	22	3.1	
≥ 20	543	26.3	129	21.1	414	28.4		16	0.8	8	0.6	8	1.1	
**Year**							0.6854							0.8881
2016	727	35.2	207	33.9	520	35.7		801	40.5	518	40.6	283	40.4	
2017	650	31.4	192	31.5	458	31.4		544	27.5	347	27.2	197	28.1	
2018	690	33.4	211	34.6	479	32.9		631	31.9	411	32.2	220	31.4	

IR, insulin resistance.

^a^Three groups (white, pink, and blue) based on the International Standard Classification of Occupations codes. The inoccupation group includes homemakers.

^b^BMI: body mass index; obesity status was defined based on BMI according to the 2018 Clinical Practice Guidelines for Overweight and Obesity in Korea.

^c^Walking frequency was based on the recommended walking activity according to the physical activity guidelines in Korea.

^d^Energy intake was classified according to the Korean Nutrient Intake Criteria (2015) provided by the Ministry of Health and Welfare.

^e^Chronic disease was defined as a diagnosed disease, such as hypertension and dyslipidemia.

^f^Family history of diabetes was defined as having an immediate family member (e.g., father, mother, brother, and/or sister) with diabetes.

### Association between smoking patterns and insulin resistance

Table 2 presents the associations between smoking patterns and insulin resistance for male and female participants after adjusting for all control variables. Compared to non-smokers, men who were continuous-smokers (odds ratio [OR] = 1.74, 95% confidence interval [CI] = 1.27–2.38) and past-smokers (OR = 1.47, 95% CI = 1.03–2.09) were at an increased risk of insulin resistance. Similarly, compared to non-smokers, women who were continuous-smokers (OR = 2.01, 95% CI = 1.33–3.03) and past-smokers (OR = 1.37, 95% CI = 1.00–1.87) were at an increased risk of insulin resistance.

**Table 2:** Association between short-term smoking patterns and the triglyceride and glucose index.

Variables	High IR					
	Men			Women		
	Adjusted OR	95% CI	*p* value	Adjusted OR	95% CI	*p* value
**Short-term smoking pattern**						
Continuous-smoker	1.74	1.27–2.38	0.001	2.01	1.33–3.03	0.001
Past-smoker	1.47	1.03–2.09	0.033	1.37	1.00–1.87	0.050
Current-smoker	1.06	0.20–5.56	0.949	0.90	0.29–2.78	0.850
Non-smoker	1.00			1.00		
**Age**						
19–29	1.00			1.00		
30–39	1.78	1.18–2.68	0.006	1.90	1.09–3.32	0.024
40–49	2.61	1.61–4.24	< 0.0001	2.60	1.52–4.43	0.000
50–59	2.54	1.44–4.46	0.001	3.92	2.26–6.78	< 0.0001
60–69	1.91	1.05–3.47	0.035	3.46	1.85–6.47	< 0.0001
≥ 70	1.27	0.65–2.48	0.493	4.30	2.13–8.70	< 0.0001
**Marital Status**						
Married	1.00			1.00		
Single. widow, divorced, separated	0.81	0.58–1.14	0.221	0.84	0.60–1.16	0.284
**Educational level**						
Middle school or below	1.00			1.00		
High school	0.76	0.49–1.17	0.209	1.02	0.71–1.45	0.927
College or over	0.80	0.50–1.27	0.347	0.91	0.60–1.37	0.637
**Household income**						
Low	1.00			1.00		
Mid-low	0.84	0.55–1.29	0.429	0.76	0.52–1.12	0.170
Mid-high	1.02	0.65–1.59	0.935	0.87	0.59–1.27	0.458
High	0.83	0.54–1.29	0.405	0.72	0.48–1.07	0.105
**Region**						
Urban area	1.00			1.00		
Rural area	1.21	0.82–1.77	0.330	0.84	0.60–1.16	0.281
**Occupational categories**^**a**^						
White	0.84	0.57–1.24	0.387	1.16	0.79–1.72	0.455
Pink	0.97	0.61–1.53	0.894	0.95	0.67–1.34	0.755
Blue	0.57	0.39–0.81	0.002	1.16	0.81–1.66	0.425
Inoccupation	1.00			1.00		
**BMI**^**b**^						
Underweight or Normal < 25	1.00			1.00		
Overweight ≥ 25.0	2.92	2.19–3.88	< 0.0001	3.87	2.99–5.02	< 0.0001
**Drinking status**						
No	1.00			1.00		
Yes	1.31	0.92–1.87	0.137	1.08	0.81–1.44	0.619
**Walking frequently**^**c**^						
Inadequate	1.00			1.00		
Adequate	0.75	0.59–0.96	0.024	0.83	0.64–1.07	0.143
**Energy intake level**^**d**^						
Inadequate	1.00			1.00		
Adequate	1.03	0.80–1.34	0.803	0.79	0.62–1.01	0.059
**Chronic disease diagnosis**^**e**^						
No	1.00			1.00		
Yes	1.35	0.98–1.86	0.066	1.40	1.01–1.95	0.041
**Secondhand smoke exposure**						
No	1.00			1.00		
Yes	1.10	0.85–1.42	0.479	0.76	0.56–1.02	0.066
**Family history**^**f**^						
No	1.00			1.00		
Yes	1.10	0.79–1.53	0.591	1.02	0.76–1.37	0.893
**Pack-Year of Smoking**						
Pack-Years < 10	1.00			1.00		
10 ≤ Pack-Years < 20	1.14	0.77–1.69	0.242	2.76	1.27–6.01	0.952
≥ 20	0.99	0.69–1.41	0.068	0.83	0.27–2.51	0.604
**Year**						
2016	1.00			1.00		
2017	0.83	0.60–1.14	0.515	0.99	0.73–1.34	0.011
2018	0.76	0.56–1.02	0.943	0.93	0.69–1.24	0.738

IR, insulin resistance; OR, odds ratio; CI, confidence interval.

^a^Three groups (white, pink, and blue) based on the International Standard Classification of Occupations codes. The inoccupation group includes homemakers.

^b^BMI: body mass index; obesity status was defined based on BMI according to the 2018 Clinical Practice Guidelines for Overweight and Obesity in Korea.

^c^Walking frequency was based on the recommended walking activity according to the physical activity guidelines in Korea.

^d^Energy intake was classified according to the Korean Nutrient Intake Criteria (2015) provided by the Ministry of Health and Welfare.

^e^Chronic disease was defined as a diagnosed disease, such as hypertension and dyslipidemia.

^f^Family history of diabetes was defined as having an immediate family member (e.g., father, mother, brother, and/or sister) with diabetes.

Table 3 presents the results of subgroup analyses stratified by the independent variable. Compared to non-smokers, male participants in the drinking group had an increased risk of insulin resistance in both the continuous-smoker and past-smoker groups (OR = 2.08, 95% CI = 1.53–2.64 and OR = 1.80, 95% CI = 1.23–2.64, respectively); female participants in the drinking group had an increased insulin resistance risk in the continuous-smoker group (OR = 1.98, 95% CI = 1.25–3.13).

**Table 3:** Subgroup analysis stratified by independent variables.

Variables	High IR									
	Short-term smoking pattern									
	Non-smoker	Continuous-smoker			Past-smoker			Current-smoker		
	OR	OR	95% CI	*p* value	OR	95% CI	*p* value	OR	95% CI	*p* value
**Men**										
**BMI**^**a**^										
Underweight or Normal < 25	1.00	1.84	1.31–2.58	0.001	1.35	0.89–2.03	0.153	2.11	0.28–15.92	0.469
Overweight ≥ 25.0	1.00	2.23	1.33–3.76	0.003	2.08	1.15–3.76	0.015	2.06	0.20–21.33	0.544
**Drinking status**										
No	1.00	2.10	0.83–5.31	0.117	0.94	0.42–2.12	0.878	–	–	–
Yes	1.00	2.08	1.53–2.64	< 0.0001	1.80	1.23–2.64	0.003	1.68	0.32–8.80	0.541
**Walking frequently**^**b**^										
Inadequate	1.00	2.42	1.55–3.79	0.001	1.34	0.83–2.16	0.229	4.61	0.38–56.11	0.229
Adequate	1.00	1.76	1.19–2.59	0.004	1.79	1.12–2.87	0.015	1.38	0.34–5.70	0.653
**Energy intake level**^**c**^										
Inadequate	1.00	1.88	1.26–2.80	0.002	1.30	0.85–2.00	0.221	1.32	0.30–5.74	0.709
Adequate	1.00	2.10	1.33–3.32	0.001	2.21	1.28–3.83	0.004	4.85	0.33–70.71	0.247
**Women**										
**BMI**^**a**^										
Underweight or Normal < 25	1.00	1.70	1.02–2.82	0.040	1.42	0.99–2.03	0.058	1.34	0.35–5.18	0.671
Overweight ≥ 25.0	1.00	2.03	0.86–4.78	0.106	1.01	0.61–1.69	0.963	2.33	0.42–13.00	0.333
**Drinking status**										
No	1.00	0.66	0.24–1.78	0.410	1.35	0.84–2.17	0.214	0.59	0.14–2.40	0.455
Yes	1.00	1.98	1.25–3.13	0.004	1.36	0.95–1.97	0.097	3.25	0.92–11.52	0.067
**Walking frequently**^**b**^										
Inadequate	1.00	2.26	1.13–4.51	0.021	1.33	0.89–1.98	0.162	1.81	0.56–5.90	0.324
Adequate	1.00	1.58	0.93–2.67	0.088	1.26	0.81–1.96	0.301	0.84	0.08–8.75	0.884
**Energy intake level**^**c**^										
Inadequate	1.00	1.62	0.99–2.66	0.053	1.13	0.80–1.58	0.489	1.45	0.39–5.41	0.581
Adequate	1.00	1.91	0.85–4.30	0.119	1.77	1.05–2.98	0.032	2.35	0.40–13.81	0.344

IR, insulin resistance; OR, odds ratio; CI, confidence interval.

^a^BMI: body mass index; obesity status was defined based on BMI according to the 2018 Clinical Practice Guidelines for Overweight and Obesity in Korea.

^b^Walking frequency was based on the recommended walking activity according to the physical activity guidelines in Korea.

^c^Energy intake was classified according to the Korean Nutrient Intake Criteria (2015) provided by the Ministry of Health and Welfare.

Obesity affected the risk of insulin resistance in male continuous-smokers and past-smokers (OR = 2.23, 95% CI = 1.33–3.76 and OR = 2.08, 95% CI = 1.15–3.76, respectively). Among women, relative to participants with normal weight, underweight participants who were continuous-smokers had the highest risk of insulin resistance (OR = 1.70, 95% CI = 1.02–2.82). Additionally, the risk of insulin resistance was the highest in the continuous-smoker group among men who rarely walked (OR = 2.42, 95% CI = 1.55–3.79) and among women who walked sufficiently (OR = 2.26, 95% CI = 1.13–4.51). Relative to non-smokers, male continuous-smokers and past-smokers with adequate energy intakes had an increased risk of insulin resistance (OR = 2.10, 95% CI = 1.33–3.32 and OR = 1.79, 95% CI = 1.12–2.87, respectively); for women, this association was observed in the past-smoker group (OR = 1.77, 95% CI = 1.05–2.98).

## Discussion

Several previous studies have examined the relationship between smoking and insulin resistance using self-reported data; however, studies on this relationship using biomarkers remain rare. Therefore, this study is one of the few studies to investigate the relationship between smoking patterns and insulin resistance using biomarkers.

Our study found that NNAL and cotinine concentrations in short-term smoking patterns were associated with insulin resistance risk in continuous- and past-smokers who met the smoking criteria. We also found that continuous smoking was significantly associated with the highest risk of insulin resistance in both men and women. However, no association was found between current smokers and insulin resistance; in this group, the smoking criteria were based only on cotinine levels. This suggests that groups with short smoking durations of around 16–20 h could be protected from complications, such as insulin resistance, by applying smoking cessation guidelines and practices.

The findings of the present study are consistent with those of previous studies, despite the use of different data sources^13^. Furthermore, our findings indirectly support those of prior studies regarding a dose–response relationship between smoking and insulin resistance^12,24^. Previous studies have shown that the amount and duration of smoking increase the risk of insulin resistance in a dose-dependent manner^24^. This finding may be due to hormonal changes associated with smoking. Moreover, smoking may induce insulin resistance directly, owing to its effect on abdominal obesity, which may partly occur due to nicotine absorption during smoking^9^. Another possible mechanism involves the smoking-triggered secretion of hormones such as cortisol, catecholamines, and growth hormones, which oppose the effects of insulin. These hormones increase lipolysis, subsequently increasing free fatty acid release and impairing endothelial function, which may contribute to insulin resistance^12^. Finally, smoking is negatively associated with adiponectin levels in a dose–response manner^25^. Therefore, these mechanisms indirectly support the association between continuous and past smoking (representing continuous smoking for > 40 days in our sample) and the risk of insulin resistance.

The stratified subgroup analysis we conducted revealed that continuous-smokers and past-smokers were at an increased risk of insulin resistance; specifically, men with a high body mass index (BMI) had an OR that was more than two-fold higher than that of non-smokers. Previous studies have suggested that smoking may cause insulin resistance by triggering processes associated with fat accumulation in the abdomen and increasing the waist-to-hip circumference ratio^9^. In addition, an increased body fat percentage has been shown to increase blood levels of non-esterified fatty acids, glycerol, hormones, pro-inflammatory cytokines, and other factors involved in the development of insulin resistance^26^, suggesting that a high BMI may increase the insulin resistance risk. Additionally, in both male and female participants, continuous-smokers with unhealthy behaviors, such as alcohol intake and lack of exercise, had a more than two-fold higher risk of insulin resistance than their counterparts. This finding supports those from previous studies on the association between unhealthy behaviors, including alcohol consumption and smoking, and serious metabolic abnormalities^27^, including insulin resistance. Moreover, continuous-smokers and past-smokers with adequate energy intakes had a two-fold higher risk of insulin resistance than their counterparts. In this study, energy intake was stratified into categories defined by the Korean nutrient intake standards^28^. However, given that smokers consume fewer essential nutrients such as vitamins, calcium, and potassium than non-smokers, it is likely that smokers meet their energy requirements by eating foods that adversely affect insulin resistance^29^. These findings may account for the increase in the insulin resistance risk that we observed in continuous-smokers and past-smokers. Further studies on the relationships between nutrient intake, smoking, and insulin resistance are required.

This study had several limitations. First, the cross-sectional study design precludes any meaningful conclusions about causality. Second, although we estimated smoking exposure and insulin resistance using urine and blood samples, respectively, data on the remaining variables were obtained from the Korea National Health and Nutrition Examination Survey (KNHANES VII) data, which were based on self-reported information; consequently, some of the estimates used may have been subject to recall bias. Third, participants with type 2 diabetes mellitus were excluded to help control for confounding factors that could affect insulin resistance; nevertheless, this restriction may have obscured or reduced the association between exposure to smoking and the risk of insulin resistance. Fourth, the study sample size was relatively small, specifically the size of the current-smoker group; this limitation was associated with the data source, whereby only half of the total sample was randomly investigated for NNAL and cotinine levels^30^. However, the KNHANES survey provides data that are nationally representative and inclusive of biomarker information, whereas previous studies did not examine these parameters. Therefore, to support our findings, future studies using larger sample sizes are required.

This study had various strengths. First, this study was based on biomarkers, in contrast to previous studies based on self-reported data. Second, this study utilized nationally representative data from the Republic of Korea, allowing us to evaluate the association between smoking patterns and insulin resistance using high-quality information; the influence of both recall bias and measurement bias on the findings is likely to be small. Finally, some previous studies have used cotinine levels to estimate smoking exposure. Herein, we also included NNAL concentrations; NNAL has a long half-life, contributing to the analysis of smoking patterns.

In conclusion, this study showed that long-term smokers (≥ 40 days) were at an increased risk of insulin resistance in short-term smoking patterns. Our findings regarding short-term smokers (16–20 h) suggest that smoking cessation may protect against complications such as insulin resistance. Therefore, there is a need to educate first-time smokers about the health benefits of quitting smoking.

## Methods

This study was based on data collected by the 2016–2018 KNHANES VII. The KNHANES comprises three parts: health surveys, health check-ups including laboratory tests, and nutrition surveys. The KNHANES is a nationwide population-based cross-sectional survey that has been conducted annually since 1998, under the direction of the Korea Centers for Disease Control and Prevention (KCDC) of the Ministry of Health and Welfare, to accurately assess the population’s health and nutritional status^31^. The KNHANES was approved by the Institutional Review Board of the KCDC, and written informed consent was obtained from all survey participants. This study adhered to the doctrine of the Declaration of Helsinki for Biomedical Research.

**Figure 1:**
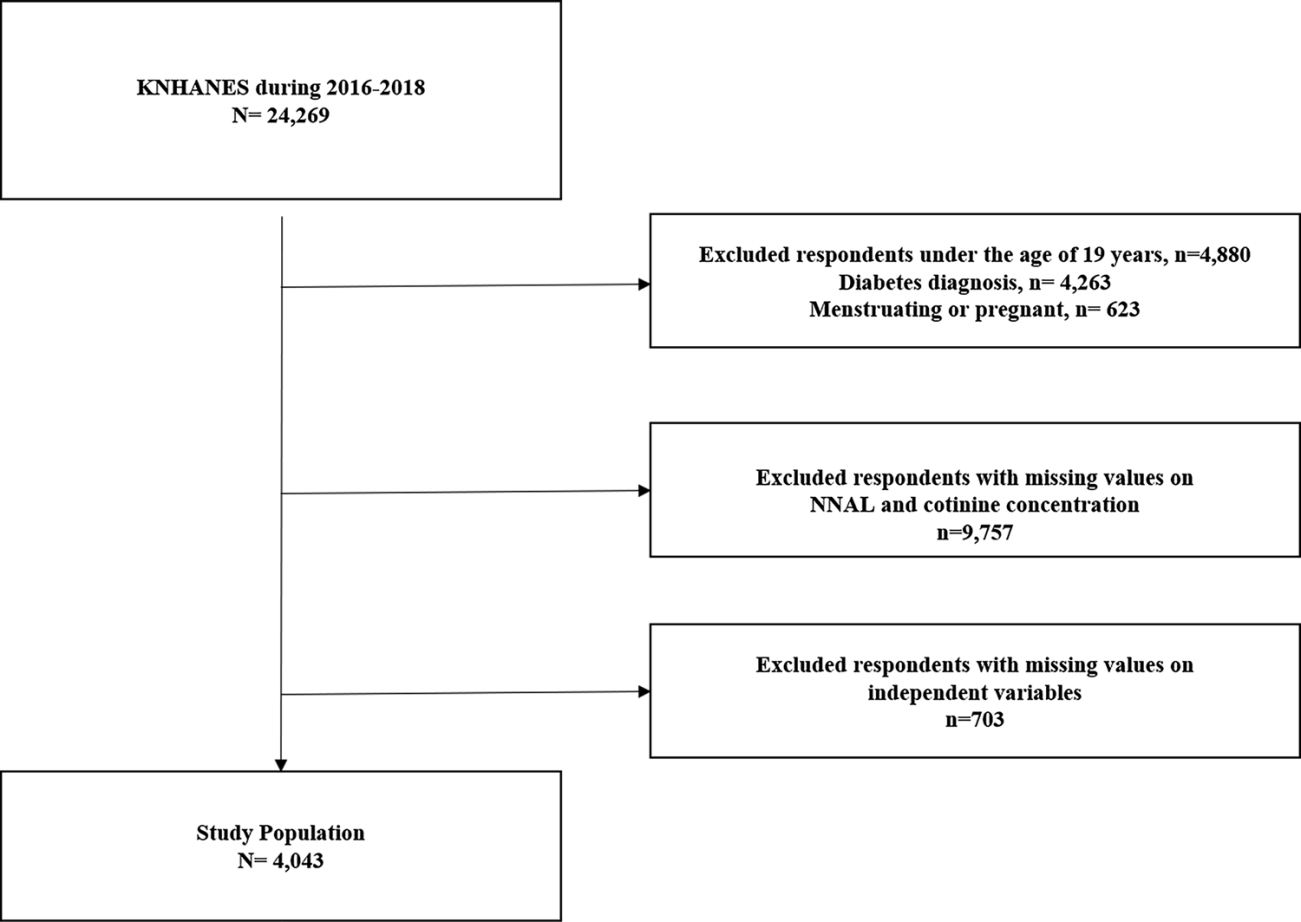
Schematic diagram of the study eligibility.

The total number of respondents during 2016–2018 was 24,269. As the KNHANES does not evaluate smoking behavior in participants younger than 19 years, data on this age group were excluded (N = 4880). In addition, we excluded participants with diabetes mellitus and those who were either menstruating or pregnant at the time of data collection (N = 4886). Finally, participants with missing values for NNAL, cotinine, and other independent variables were excluded (N = 10,460). Thus, a total of 4043 participants (2067 men and 1976 women) were evaluated (Fig. 1).

## Variables

The dependent variable in this study was the TyG index, a product of fasting triglyceride and glucose blood levels, which helps assess insulin resistance^32^. In the KNHANES data, fasting (starting after 7 p.m. the day before the survey) blood samples were provided for testing. The TyG index was calculated using the formula, ln(triglyceride [mg/dL] × fasting blood glucose [mg/dL]/2), and expressed on a logarithmic scale^5^.

We defined short-term smoking patterns by measuring the concentrations of NNAL and cotinine. Spot urinary samples were collected for urinary NNAL and cotinine at the time of health checkup. Urine cotinine was examined in all subjects aged 6 years and above at the health checkup, and NNAL was randomized in half of the health checkup subjects^30^. Fresh urine samples were collected and immediately underwent routine urinalysis, and the remaining aliquots were stored at –20 °C until the analysis of cotinine and NNAL^33^. Urine concentrations of cotinine and total NNAL (free NNAL plus NNAL-glucuronide) were analyzed by liquid chromatography-tandem mass spectrometry (LC–MS/MS) using Agilent 1100 Series API 4000 (AB Sciex, Foster City, CA, USA) and Agilent 1200 Series Triple Quadrupole 5500 (AB Sciex, Foster City, CA, USA), respectively^34,35^. The limit of detection was 0.27399 ng/mL for cotinine and 0.1006 pg/mL for NNAL^30,36^.

In this study, NNAL and cotinine concentrations were used to classify the participants into smoking and non-smoking groups using the smoking concentration standards of the KCDC (2.13 pg/mL and 50 ng/mL for NNAL and cotinine, respectively)^37^. We defined “short-term smoking pattern” based on half-life values (i.e. 18–24 h, 40 days for cotinine and NNAL)^23,38^. A "Continuous smoker" was defined as a participant who met both smoking criteria for 18–24 h and 40 days. Thus, both NNAL and cotinine concentrations are participants who meet smoking criteria. "Current-smokers" were defined as those who did not meet the 40 days smoking criteria but met the 18–24 h smoking criteria. Therefore, participants who did not meet the NNAL smoking concentrations criteria but did meet the Cotinine smoking concentrations criteria. "Past-smokers" were defined as those who met the 40 days smoking criteria but not the 18–24 h smoking criteria. Therefore, participants who meet the NNAL smoking concentrations criteria but not the cotinine smoking concentrations criteria. "Non-smoker" was defined as a person who did not meet both smoking criteria for 18–24 h and 40 days. Therefore, both NNAL and cotinine concentrations do not meet the smoking criteria (Fig. S1).

Potential confounding variables included sociodemographic and health-related characteristics and the study year. Sociodemographic characteristics included age, marital status, educational level, household income, region, and occupation. Health-related characteristics included BMI, drinking status, walking frequency, energy intake level, chronic disease diagnosis, secondhand smoking exposure, family history of diabetes, and pack-year estimates.

### Statistical analyses

Before the analysis, we excluded cases where there was no response to the variables required for the study (Fig. 1). Therefore, in this study, all estimates were calculated using sample weights assigned to the study participants. The sample weights were constructed by the KNHANES to represent the population in the Republic of Korea while accounting for the complex survey design and survey non-response^31^. Additionally, we performed a pre-analysis to classify participants into “low” and “high” insulin resistance groups. We analyzed the TyG index using receiver operating characteristic curves to estimate valid cut-off values for impaired fasting glucose levels, and the effective cut-off values for the TyG index were 8.3878 and 8.60248 for men and women, respectively; these were similar to previously reported values^39^ and were used in this study. A univariate linear regression analysis was conducted to investigate the general characteristics of the study population. Multiple regression analyses were performed and adjusted for covariates to analyze the association between smoking patterns and insulin resistance. Further, subgroup analyses were performed with multiple linear regression models stratified by sex to investigate the associations of BMI, drinking status, walking frequency, and energy intake levels with insulin resistance. ORs and 95% CIs were calculated to compare non-smokers to continuous-smokers, current-smokers, and past-smokers. All statistical analyses were performed using SAS software version 9.4 (SAS Institute, Inc., Cary, NC, USA). Findings were considered significant at *P* values  < 0.05.

## Supplementary Information

Supplementary Information.

## Data Availability

The datasets generated or analyzed during the current study (Korea National Health and Nutrition Examination Survey 2016–2018) are available at https://knhanes.kdca.go.kr/knhanes/eng/index.do.
