# Supplementary material for: Short-term smoking increases the risk of insulin resistance
Source: Sci Rep. 2022 Mar 3;12:3550. doi: 10.1038/s41598-022-07626-1 (PMC8894492; doi:10.1038/s41598-022-07626-1)
Supplement: Supplementary file 1 — Supplementary Information. [file 41598_2022_7626_MOESM1_ESM.docx]

**Supplementary data**


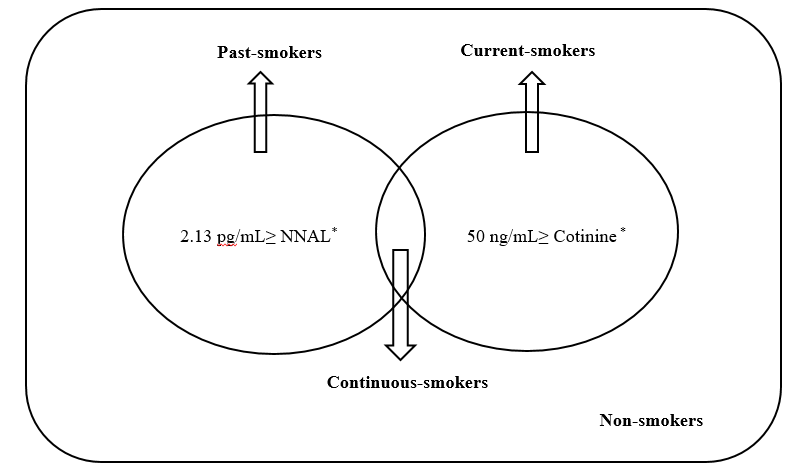


^*^ Criteria for smoking concentration of NNAL and Cotinine

Figure S1. Venn-Diagram of the Short-term smoking pattern. Short-term smoking patterns were classified according to whether the concentrations of NNAL and cotinine met the smoking criteria. The half-life of cotinine is approximately 18 to 24 h and the half-life of NNAL is approximately 40 days. **Continuous-smokers** : "Yes" based on smoking concentration criteria in both NNAL and Cotinine; **Current-smokers** : "No" based on NNAL smoking concentration criteria, "Yes" based on Cotinine smoking criteria; **Past-smokers** : "Yes" based on NNAL smoking concentration criteria, "No" based on Cotinine smoking criteria; **Non-smokers** : "No" based on smoking concentration criteria in both NNAL and Cotinine.
